# The Effect of Hypoxia on Relative Biological Effectiveness and Oxygen Enhancement Ratio for Cells Irradiated with Grenz Rays

**DOI:** 10.3390/cancers14051262

**Published:** 2022-02-28

**Authors:** Chun-Chieh Chan, Fang-Hsin Chen, Kuang-Lung Hsueh, Ya-Yun Hsiao

**Affiliations:** 1 Department of Computer Science and Information Engineering, National Formosa University, Yunlin 632301, Taiwan; ccchan@nfu.edu.tw; 2 Department of Medical Imaging and Radiological Sciences, Chang Gung University, Taoyuan 33302, Taiwan; fanghsinchen@mail.cgu.edu.tw; 3 Radiation Biology Research Center, Institute for Radiological Research, Chang Gung University, Taoyuan 33302, Taiwan; 4 Department of Radiation Oncology, Chang Gung Memorial Hospital-Linkou Branch, Taoyuan 33305, Taiwan; 5 Institute of Biomedical Sciences, Academia Sinica, Taipei 11529, Taiwan; renshiue@thu.edu.tw; 6 Department of Animal Science and Biotechnology, Tunghai University, Taichung 407224, Taiwan; 7 Department of Radiology, Chung Shan Medical University Hospital, Taichung 40201, Taiwan; 8 Department of Medical Imaging and Radiological Sciences, Chung Shan Medical University, Taichung 40201, Taiwan

**Keywords:** double strand break, cell survival, hypoxia, relative biological effectiveness, oxygen enhancement ratio, Grenz rays

## Abstract

**Simple Summary:**

Grenz rays are low energy X-rays and have been used for dermatological radiotherapy for decades. This study calculates the relative biological effectiveness (RBE) and oxygen enhancement ratio (OER) for DNA double-strand break (DSB) induction and cell survival under hypoxic conditions for Grenz-ray therapy (GT). The RBE values for cell survival for cells irradiated by 15 kV, 10 kV and 10 kVp and titanium K-shell X-rays (4.55 kV) relative to ^60^Co γ-rays are 1.0–1.6 at an aerobic condition (21% O_2_) and further increase to 1.2–2.1 in conditions of severe hypoxia (0.1% O_2_). The OER values for DSB induction relative to ^60^Co γ-rays are ~2.4 for GT, but the OER for cell survival is 2.8–2.0 as photon energy decreases from 15 kV to 4.55 kV.

**Abstract:**

Grenz-ray therapy (GT) is commonly used for dermatological radiotherapy and has a higher linear energy transfer, relative biological effectiveness (RBE) and oxygen enhancement ratio (OER). GT is a treatment option for lentigo maligna and lentigo maligna melanoma. This study aims to calculate the RBE for DNA double-strand break (DSB) induction and cell survival under hypoxic conditions for GT. The yield of DSBs induced by GT is calculated at the aerobic and hypoxic conditions, using a Monte Carlo damage simulation (MCDS) software. The RBE value for cell survival is calculated using the repair–misrepair–fixation (RMF) model. The RBE values for cell survival for cells irradiated by 15 kV, 10 kV and 10 kVp and titanium K-shell X-rays (4.55 kV) relative to ^60^Co γ-rays are 1.0–1.6 at the aerobic conditions and moderate hypoxia (2% O_2_), respectively, but increase to 1.2, 1.4 and 1.9 and 2.1 in conditions of severe hypoxia (0.1% O_2_). The OER values for DSB induction relative to ^60^Co γ-rays are about constant and ~2.4 for GT, but the OER for cell survival is 2.8–2.0 as photon energy decreases from 15 kV to 4.55 kV. The results indicate that GT results in more DSB induction and allows effective tumor control for superficial and hypoxic tumors.

## 1. Introduction

Grenz rays are soft X-rays with energy less than 20 kV [1], used in what is known as Grenz-ray therapy (GT) [2]. GT is used for dermatological treatment in allergic diseases, such as eczema, contact dermatitis and psoriasis [2,3]. GT can also be used as a treatment option for lentigo maligna (LM) and lentigo maligna melanoma (LMM), with high local control rates of approximately 90% or more [4,5,6,7,8,9,10]. In contrast to conventional X-ray therapy (XT), GT has minor adverse effects, such as erythema and pigmentation [5,11]. However, GT has been reported to induce carcinoma [11,12].

The mechanism for DNA damage induction for cells irradiated by ionizing radiations involves direct and indirect actions [13]. For low linear energy transfer (LET) radiations, such as photons and protons, about two-thirds of DNA damage induction result from indirect actions, mostly from hydroxyl free radicals (OH) [13]. These radicals result from the cascade of secondary electrons in the water molecule and subsequently diffuse into surrounding DNA, where they react to form strand breaks or base damages [13]. Under hypoxic conditions, these secondary free radicals react with endogenous thiols, such as glutathione, which restores the original undamaged structure by chemical repairing [14]. In the presence of oxygen, oxygen competes with the thiols and reacts with the DNA radical to produce a non-restorable organic peroxide, which subsequently reacts to produced DNA-OOH and chemically fixes the damage. This is the oxygen fixation hypothesis [13].

The relative biological effectiveness (RBE) depends on photon energy. The RBE of low energy photons increases as energy decreases [15,16,17]. The RBE for double-strand break (DSB) induction relative to ^60^Co γ-rays increases from 1.4 to 2.7 as the photon energy decreases from 4.55 keV to 280 eV [17]. The RBE values for cell inactivation for Chinese hamster ovary V79 cells increase from 1.5 to 3.5 as the photon energy decreases from 8 keV to 280 eV [18], but these values vary with cell type and laboratory [19]. In general, Grenz rays are up to six times more effective than 250 kVp X-rays in regard to DSB yield, cell death and mutations [19,20,21,22].

GT has been used for radiation treatment for decades [23,24]. The DNA profiles and RBE values are required to determine the dose prescription for clinical treatment. RBE values for cell survival can be predicted by mathematical models, such as the local effect model [25,26], the microdosimetric-kinetic model [27,28,29,30], the NanOX model [31] and the DSB-based repair–misrepair–fixation (RMF) model [32,33,34]. The RMF model is used to make predictions of cell survival and RBE for various human cell lines [35] and has been used for biologically optimized treatment planning [36,37].

The measured RBE values for soft X-rays under hypoxic conditions are available [17,18,38,39,40], but to our best knowledge, no other simulation estimates the RBE values for GT under hypoxic conditions. A previous study by Hsiao and Stewart [41] has developed a fast algorithm to estimate the RBE for DSB induction for photon beams as low as 27 kV (^125^I gamma rays) at an aerobic condition (21% O_2_) using the fluence of secondary electrons. This study extends this algorithm to estimate the RBE for DSB induction for photon beams with lower energy, ranging from 4.55 kV to 15kV. Furthermore, the RBE for cell survival using the RMF model is determined for different oxygen concentrations, allowing the prediction of RBE for cell survival for hypoxic tumors.

## 2. Materials and Methods

### 2.1. Irradiation Geometries and Materials

A single-cell cylindrical geometry was used to simulate the low-energy photon irradiations, which has been used by others [41,42]. The cylinder was designed with a radius of 10 µm and a height of 20 µm, including a 4 μm mylar foil (density 1.4 g/cm^−3^) between the photon beam and the cell. The single-cell geometry was approximated by water’s density (1.0 g/cm^−3^), as used in a previous study [41].

### 2.2. Determination of Secondary-Electron Fluence

The PENCYL program in the version 2011 penetration and energy loss of positrons and electron (PENELOPE) Monte Carlo code [43,44,45] was used to calculate the fluence of secondary electrons passing over single cells. PENELOPE has been showed to produce photon and electron spectrum for low-energy X-rays [46]. All PENCYL simulations were performed with at least 2 × 10^8^ source particle histories, which ensure that standard deviations of the secondary electron energy fluence are accurate to within about 10%. Comparisons of the depth dose for titanium K-shell photons (4.55 kV), which are the lowest photon energies studied in this work, at the top (~4.1 μm) and the bottom part (~20 μm) of the water medium in the cell differ by less than 12%, which implies that the absorbed dose is constant everywhere within the water medium for the considered irradiation geometry. All primary and secondary electrons were followed down to 50 eV [47]. The electron transport processes were simulated using PENELOPE, and the simulations were controlled by the parameters C_1_, C_2_, W_CC_ and W_CR_ whose definitions and roles were explained previously [48]. The values of parameters in this study were set as C_1_ = 0.00, C_2_ = 0.01, W_CC_ = 10 eV and W_CR_ = 10 eV. The spectrum for 10 kVp X-rays was derived from PENELOPE applying the geometry (Chromium (Cr) anode; 0.2 mm Cr filter) used in a previous study [49].

### 2.3. Monte Carlo Damage Simulation (MCDS)

MCDS provides the DNA damage yields and spatial information for cells irradiated with photons and ions up to ^56^Fe at various oxygen concentrations [50,51,52]. The MCDS employs clustering algorithms to estimate DNA damage induction. Specifically, MCDS generates random numbers of damage configurations within one cell and simulates the process of DNA damage in two main steps: (1) the initial damage within a cell is randomly distributed in a DNA segment and (2) this distribution of DNA damage in the segment is subdivided into lesions [50]. This damage grouping process is an arbitrary procedure of identifying a subset of the elementary damages in the DNA segment. That is, two elementary damages separated by at least *N_min_* = 9 base pairs are treated as different lesions. It is a fast algorithm compared to detailed but computationally expensive track structure simulations [53,54,55,56].

Types of DNA damage can be classified as base damage (BD), single-strand break alone (SSB), DSB alone, two or more strand breaks on the same strand (SSB^+^), two or more strand breaks on opposite strands but separated more than ten base pairs and do not constitute a DSB (2SSB), DSBs with additional break(s) on a strand within ten base pairs (DSB^+^) and more than one DSB within ten base pairs (DSB^++^). The total SSB refers to the total number of single-strand breaks (SSB, SSB^+^ and 2SSB), and the total DSB refers to the total number of double-strand breaks (DSB, DSB^+^ and DSB^++^).

### 2.4. Calculation of DSB Induction

For the uniform irradiation conditions assumed in the MCDS, the absorbed dose rate is equal to the product of the expected number of primary charged particles per second of energy *E* passing through the target $\overset{\cdot }{\nu }(E)$ times the frequency–mean specific energy per event ${\bar{z}}_{F}$ [57], i.e., $\overset{\cdot }{D}(E)=\overset{\cdot }{\nu }(E){\bar{z}}_{F}$. ${\bar{z}}_{F}$, and the frequency–mean specific energy for a spherical target composed of water with diameter *d* is given by [57]:

(1) $${\bar{Z}}_{F}=0.204\frac{LET}{d^{2}}\left(\frac{\mathrm{kev}}{\mathrm{\mu m}}\right)$$

The DSB inductions for ultrasoft X-rays and ^60^Co γ-rays were calculated with the dose-weighted formula [41]:

(2) $$Y=\frac{\int_{0}^{\infty }dEY(E)\Phi (E){\mathrm{LET}}_{\infty }(E)}{\int_{0}^{\infty }dE\Phi (E){\mathrm{LET}}_{\infty }\left(E\right)}$$

where *Y*(*E*) represents the yield of the DSB induction per Gy per gigabase pairs (per Gy per Gbp) with the secondary electrons of energy *E*. Φ(*E*) is the energy fluence of secondary electrons of energy *E* that are generated in the cell medium from ultrasoft X-rays or ^60^Co γ-rays photon interactions. The sources of the value for unrestricted *LET* (stopping power) were described previously [41].

### 2.5. Statistical Methods

The standard error of the mean (SEM) for the DNA damage yields reported in Tables 1–5 by MCDS was calculated by the formula: $SEM=\frac{s}{\sqrt{n}}$, where *s* is the standard error and *n* is the number of samples. *n* is set as 10,000 for all DNA damage yields reported in this study. The line of bestfit (Figure 3B) was obtained using the “Trend line” function in Microsoft Excel software. All analyses for *p* values were performed by Student’s *t*-test. *p* value < 0.05 was considered statistically significant.

### 2.6. RMF Model

An RMF model [32] was developed to better link DSB induction to reproductive cell death. In the case of low dose and low dose rate, the *α* and *β* coefficients of the linear-quadratic (LQ) model [54] can be derived from the RMF mode and are expressed as:

(3) $$\alpha =\left[1-f_{R}\left(1-\theta \right)\right]\sum +\kappa {\bar{z}}_{F}{\left(f_{R}\sum \right)}^{2}$$

(4) $$\beta =\left(\kappa /2\right){\left(f_{R}\sum \right)}^{2}$$

where $f_{R}$ is the fraction of the initial DSBs that are potentially rejoinable and defined below as:

(5) $$f_{R}=\frac{1}{\sum }\sum_{i=2}^{j-1}\sum_{i}$$

The parameter *j* defines the least number of lesions per DSB that cannot be rejoined, ∑ is the total number of DSBs per cell per Gy and $\sum_{i}$ is the expected number of DSBs per cell per Gy, which is composed of exactly *i* lesions. ${\bar{Z}}_{F}$, the frequency–mean specific energy, is described above. The diameter was set as 8 μm, which was the typical size of V79 Chinese hamster cells used in many studies [58]. θ is defined as the fraction of DSBs that undergo lethal first order misrepair and damage fixation, and κ is defined as the fraction of initial DSBs that undergo pairwise damage interactions [32]. Parameter *j* ~13 is estimated for the survival data of V79 cells [17] at an aerobic condition and a moderate hypoxic condition (2% O_2_) [59]. At an oxygen concentration of 0.1%, the value for parameter *j* ~10 is used to fit to the measured OER data. All calculations for RBE values for cell survival at a normal oxygen condition (21% O_2_) or at a moderate hypoxic condition (2% O_2_) use values of θ = 5.79 × 10^−3^ and κ = 5.59 × 10^−5^, as suggested in a previous study [32]. For a severely hypoxic condition (0.1% O_2_) [59], the calculations use values of θ = 4.25 × 10^−3^ and κ = 4.5 × 10^−5^.

### 2.7. RBE for DSB Induction and Cell Survival

The RBE is generally defined as the ratio of the dose of low *LET* reference radiation to the dose of any other radiation to cause the same level of biological effect [13]. The RBE is also interpreted as a ratio of the DSB yield, Σ, because DSB induction is linearly proportional to the absorbed dose, *D*, up to a hundred Gy under an aerobic condition (21% O_2_) [60] and a severely hypoxic condition (0.1% O_2_) [61] and are expressed as:

(6) $$RBE=\frac{D_{\gamma }}{D_{G}}=\frac{\Sigma_{G}}{\Sigma_{\gamma }}$$

Subscripts *G* and *γ*, respectively, denote Grenz rays and γ-rays. The DSB yield for ^60^Co γ-rays is the reference for all reported RBE values.

Cell survival is described in the LQ model [13]:

(7) $$S=e^{-\left(\alpha D+\beta D^{2}\right)}$$

where *S* denotes the survival fraction of cells at dose *D*, and α and β are the curve-fitting parameters. Using Equation (7) and the dose definition in Equation (6) may be shown as:

(8) $$D_{G}=\frac{1}{2}\left\{-\left(\frac{\alpha_{G}}{\beta_{G}}\right)+\sqrt{{\left(\frac{\alpha_{G}}{\beta_{G}}\right)}^{2}-\frac{4\left(\alpha_{G}/\beta_{G}\right)\ln\left(S\right)}{\alpha_{G}}}\right\}$$

(9) $$D_{\gamma }=\frac{1}{2}\left\{-\left(\frac{\alpha_{\gamma }}{\beta_{\gamma }}\right)+\sqrt{{\left(\frac{\alpha_{\gamma }}{\beta_{\gamma }}\right)}^{2}-\frac{4\left(\alpha_{\gamma }/\beta_{\gamma }\right)\ln\left(S\right)}{\alpha_{\gamma }}}\right\}$$

where $\alpha_{G}$, $\beta_{G}$, $\alpha_{\gamma }$ and $\beta_{\gamma }$ are the α and β parameters that are defined in Equations (2) and (3) for Grenz-ray and ^60^Co γ-ray exposure, respectively.

### 2.8. Oxygen Enhancement Ratio (OER)

The effect of oxygen is generally quantified as the ratio of the hypoxic dose to the aerated dose to cause the same level of biological outcome, which is termed oxygen enhancement ratio (OER) [62]. Some studies also used OER as the ratio of the biological effect, such as DSB yield, at the same dose [62,63]. Here, the OER for DSB induction is defined as the DSB yield induced under 21% O_2_ to the yield induced under 0.1% O_2_. The OER for cell survival is defined in Equation (10), where the doses *D_a_* (at an aerobic condition (21% O_2_)) and *D_h_* (at a severely hypoxic condition (0.1% O_2_)) are calculated using Equations (11) and (12), respectively, as shown below:

(10) $$OER=\frac{D_{h}}{D_{a}}$$

(11) $$D_{a}=\frac{1}{2}\left\{-\left(\frac{\alpha_{a}}{\beta_{a}}\right)+\sqrt{{\left(\frac{\alpha_{a}}{\beta_{a}}\right)}^{2}-\frac{4\left(\alpha_{a}/\beta_{a}\right)\ln\left(S\right)}{\alpha_{a}}}\right\}$$

(12) $$D_{h}=\frac{1}{2}\left\{-\left(\frac{\alpha_{h}}{\beta_{h}}\right)+\sqrt{{\left(\frac{\alpha_{h}}{\beta_{h}}\right)}^{2}-\frac{4\left(\alpha_{h}/\beta_{h}\right)\ln\left(S\right)}{\alpha_{h}}}\right\}$$

where $\alpha_{a}$, $\beta_{a}$, $\alpha_{h}$ and $\beta_{h}$ are the α and β parameters that are defined in Equations (2) and (3), respectively, under an aerobic condition (21% O_2_) and at a severely hypoxic condition (0.1% O_2_).

## 3. Results

The DNA profiles of cells irradiated by photon beams are from the DNA profiles of secondary electrons, so the results of this study are compared with those derived from published track structure simulations. Table 1 lists the DNA profiles of cells irradiated with electron energy of 100 eV to 4500 eV. The difference between the percentages of DSB calculated by MCDS and track structure simulations decreases from 3% to 0.1% as the electron energy increases. For energy of 1500 eV, the greatest difference is in SSB and DSB^+^, of about 24% and 42%, but the values of the latter are also very small. The percentages of BD using track structure simulations are 30–5% higher than those obtained by MCDS. The percentage of SSB obtained by track structure simulations is 11–6% lower than those obtained by MCDS. BD and SSB are categorized as simpler damage, so the DNA profiles derived from these two different simulations, other than BD and SSB, are close to each other for an energy of 1500 eV or higher.

The results in Table 1 are used to simulate a cell irradiated by photon beams with energy up to 4.5 keV. The *p* values of the comparisons in SSB and DSB yields between any two groups of 100, 300, 500, 1000 and 1500 eV are less than 0.01. Most photon interactions for photon energy less than 50 keV are photoelectric interactions, so the energy of the emitted photoelectrons is equal to the photon energy minus the binding energy of the electrons. The binding energy of the electrons in the K shell of oxygen is ~543 eV [67], so the energy of photoelectrons before attenuation in the single-cell geometry is about 4 keV (4.55 − 0.55 = 4) or more. The mean electron energy (estimated by PENELOPE) for cells irradiated by single-cell geometry for 4.55 kV photons is about 2.8 keV (>1.5 keV). The deviation between the DNA profiles from track structure and MCDS increases as electron energy decreases. For example, the percentage of DSB induced by 100 eV electrons using track structure is 1.39%, but the value using MCDS is 4.72%. The deviation in the total DSB yields reduces to 5% when the energy increases to 500 eV. For 4.55 keV photons (this work), ~0.4% of electrons are ~100 eV, ~4% are 100–500 eV and a total 9% are 1000 eV below. The maximum deviation in the total DSB yields is estimated to be 1.4 ((24.9 − 9.8) × 0.09) per Gbp per Gy, suggesting the DSB yield using MCDS may be ~13% (1.4/10.8) overestimated to the value that is derived using track structure simulations.

Table 2 shows the DNA profiles of 4.55 kV, 10 kVp, monochromatic 10 kV and 15 kV X-rays. The yield of more complicated types of DNA damage, such as DSB^+^ and DSB^++^, increases 7–62% as photon energy decreases, so there is a 21% increase in the yield of total DSBs as photon energy decreases. In contrast, the yield of simpler types of damage, such as BD and SSBs, decreases 8–11% as photon energy decreases, although the percentage of SSBs increases 4% as photon energy decreases. The increase in the yield for more complicated types of DNA damage is 8–10% the complexity of DNA damage as photon energy decreases. A comparison of the DNA profile with that for the track structure [68] for cells irradiated by 4.55 kV photons shows that the results obtained by MCDS give a more complex DNA damage spectrum, i.e., lower percentage of BD and SSB and higher percentages of DSB, DSB^+^ and DSB^++^, which is also reflected in the DNA profiles of electrons (Table 1).

Table 3 shows that the RBE for DSB induction increases from 1.1 to 1.3 as the photon energy decreases from 15 keV to 4.55 keV. The measured RBE value for DSB induction for cells irradiated by 4.55 kV photons is 1.4 [17], which is close to the value calculated using MCDS. The complexity of the DNA damage is significantly affected by indirect actions in which oxygen plays an important role for fixing DNA damages and therefore affects the complexity. The yield of DNA damage is further simulated at an oxygen concentration of 2% (hypoxic conditions, Table 4) and 0.1% (Table 5). The resulting yield of DSBs, DSBs^+^ and DSBs^++^ is less than the values at an oxygen concentration of 21%, regardless of the level of photon energy (Table 2). The percentage of BD decreases as photon energy decreases, while the percentage for other classes increases as photon energy decreases. At an oxygen concentration of 2% or 0.1% (Table 5), the RBE values for DSB induction relative to ^60^Co γ-rays are about the same for all energy levels (Table 4 and Table 5).

**Figure 1:**
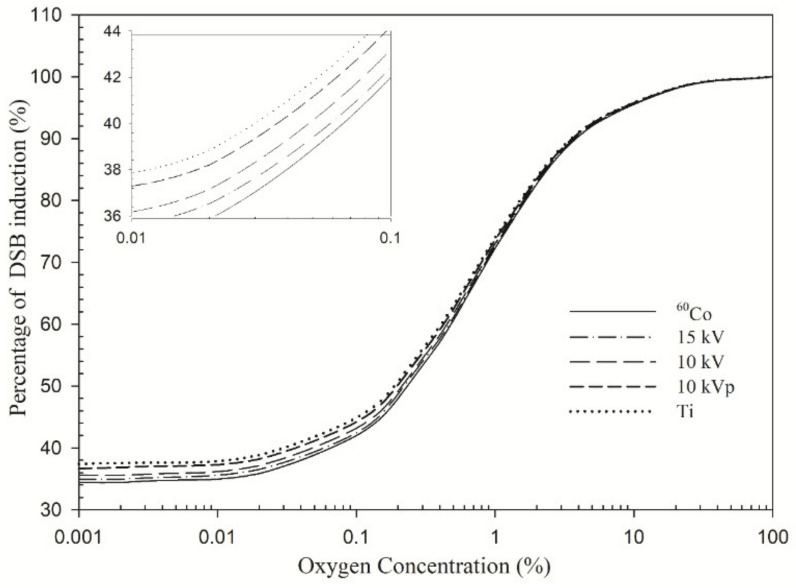
Percentage of double-strand break (DSB) induction as a function of oxygen concentration: the symbol Ti denotes titanium K-shell.

Figure 1 shows the percentage of DSB induction as a function of oxygen concentration at 0.001–100% for monoenergetic 15 kV, 10 kV, 10 kVp and 4.55 kV X-rays and ^60^Co γ-rays. All of the percentages are 35–42% within 0.001–0.1% O_2_ and gradually increase to 82% or higher as the oxygen concentration exceeds 2% O_2_. The difference between these energy levels decreases as the oxygen concentration increases.

**Figure 2:**
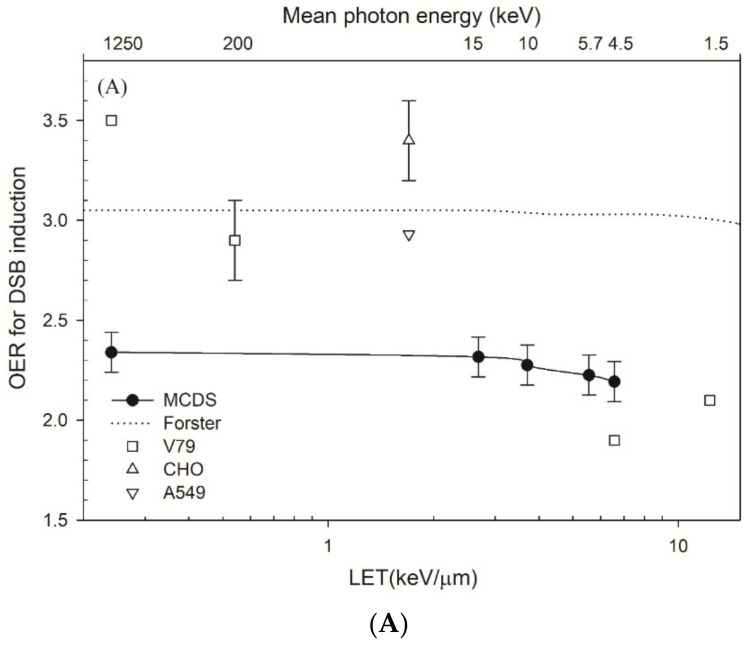

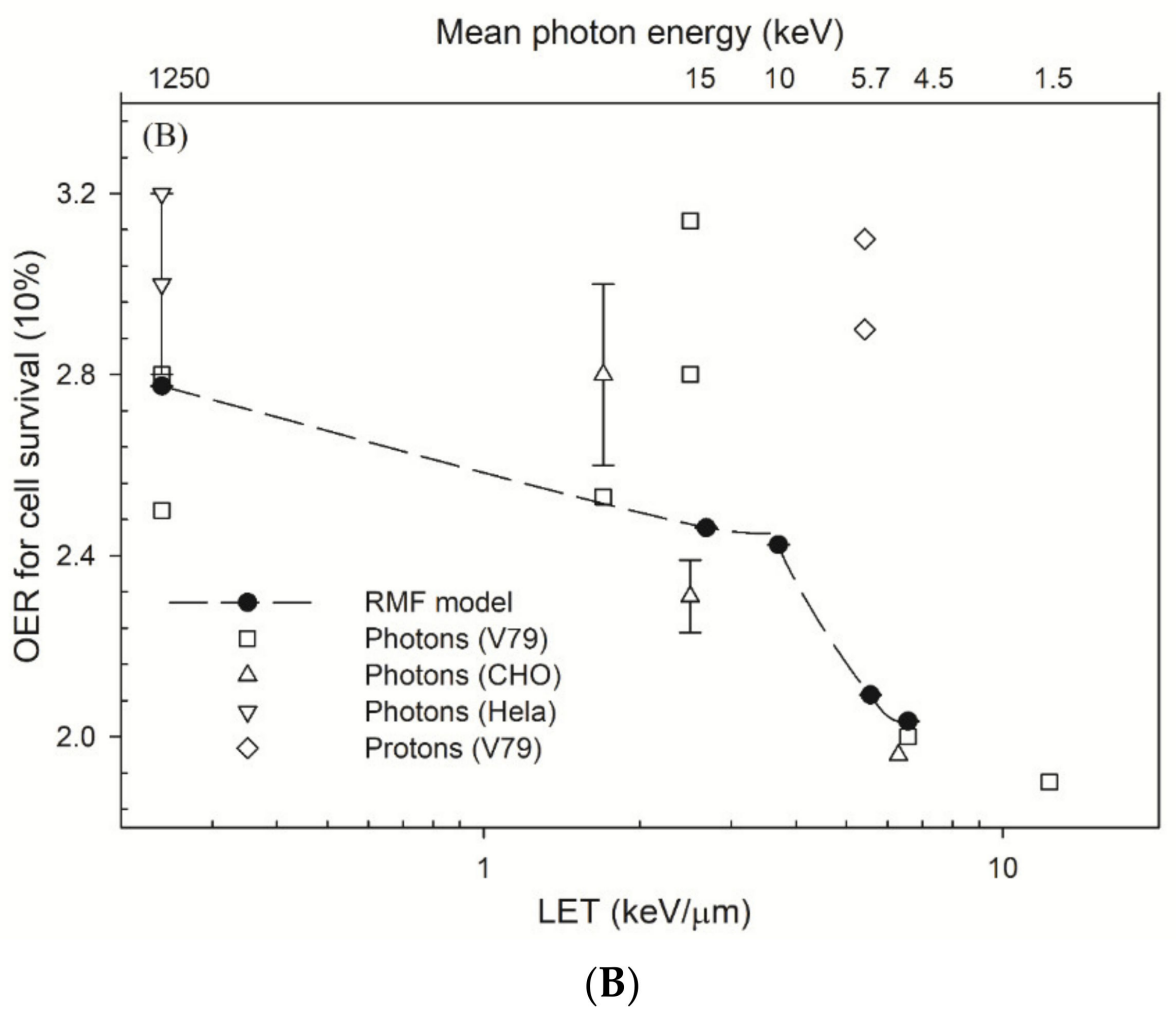
(**A**) Oxygen enhancement ratio (OER) for double-strand break (DSB) induction for cells irradiated by photons. The lines are the Monte Carlo damage simulation (MCDS) results and those for other Monte Carlo simulations [69]. The individual symbols represent the experimental OER values for V79 cells [17,61], Chinese hamster ovary (CHO) cells [38] and human non-small cell lung cancer A549 cells [70]. (**B**) OER for cell survival at 10% as a function of linear energy transfer (LET) and mean photon energy. These measured OER values are from published studies: CHO cells [38,39,71], Hela cells [72] and V79 cells irradiated with photons [17,18,40,73,74] and protons [18].

Figure 2A shows that the respective OER_DSB_ values calculated by the MCDS for ^60^Co γ-rays, monochromatic 15 kV, 10 kV, 10 kVp and 4.55 kV X-rays are 2.3–2.1 for an oxygen concentration of 0.1%. The other simulated OER value for DSB induction is ~3.0 as *LET* increases to 13.0 keV/μm [69]. The experimental data show that the OER for DSB induction for ^60^Co γ-rays, 200 kV and 4.55 kV X-rays is 3.5, 2.9, and 1.9 [17]. Figure 2B shows the OER for cell survival at 10% survival levels versus LET. There is a greater decrease in OER for cell survival than that for DSB induction. The OER for cell survival decreases from 2.8 to 2.0 as *LET* increases by 6.6 keV/μm. The experimental data show that the OER for cell survival oscillates at *LET* less than 2 keV/μm but decreases as *LET* increases from 2 to 13 keV/μm.

**Figure 3:**
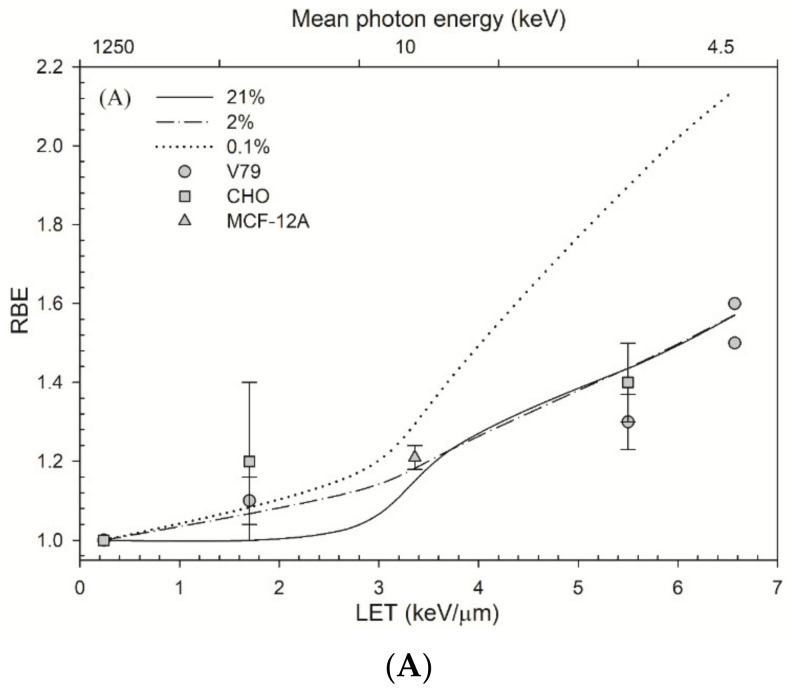

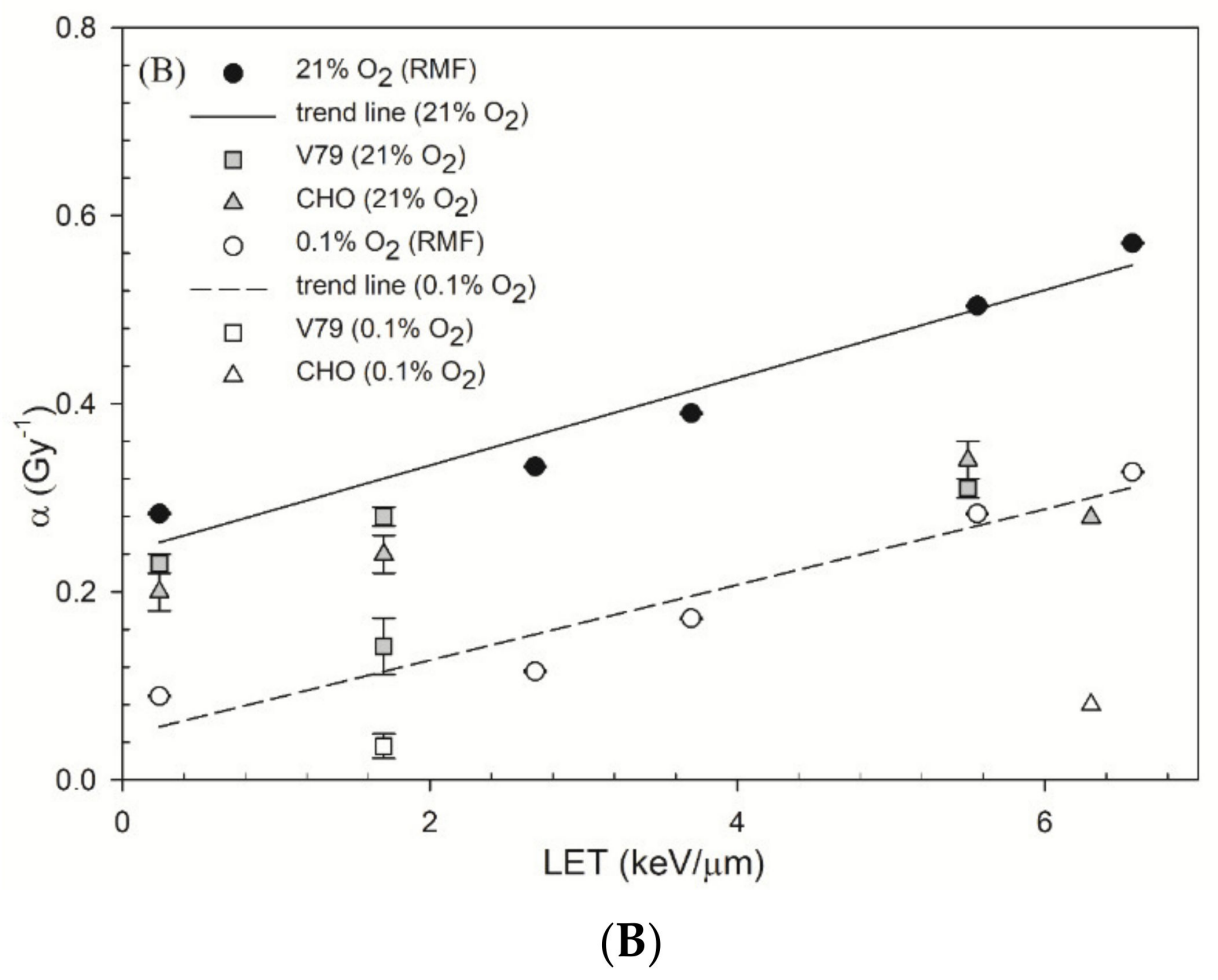
Relative biological effectiveness (RBE) values for cells irradiated by ultrasoft X-rays as a function of linear energy transfer (LET). (**A**) The repair–misrepair–fixation (RMF)-model-predicted RBE values for cell survival at 10% are plotted for oxygen concentrations of 21% (solid line), 2% (dash-dotted line) and 0.1% (dotted line). The experimental RBE values were measured at an oxygen concentration of 21% O_2_ for V79 cells [17,75,76], Chinese hamster ovary (CHO) cells [75] and human mammary epithelial cell line MCF-12A [77] after photon irradiations. (**B**)The values for parameter α using RMF model: the equations for the α value under aerobic and hypoxic conditions are, respectively, α = 0.0466 × *LET* + 0.2412 (R^2^ = 0.9443) (21% O_2_) and α = 0.0402 × *LET* + 0.0466 (R^2^ = 0.9161) (0.1% O_2_). These measured α values are from published studies: CHO cells and V79 cells irradiated with photons at an aerobic condition (21% O_2_) [61,71,75] and at a hypoxic condition (0.1% O_2_) [61,71].

Figure 3A shows that the RBE values for cell survival at an aerobic condition are 1.0–1.6, which agrees well with the measured cell survival. The RBE values at a 2% oxygen concentration are also 1.0–1.6. Under a severely hypoxic condition (0.1% oxygen concentration), the RBE value for cell survival increases from 1.2 to 2.4. Figure 3B shows there is a linear relationship between parameter α and *LET* under aerobic conditions (21% O_2_) and severely hypoxic conditions (0.1% O_2_). The MCDS-derived values for parameter α increase linearly (R^2^ = 0.9443) from 0.283 to 0.571 Gy^−1^ at an aerobic condition (21% O_2_) and also (R^2^ = 0.9161) from 0.089 to 0.327 Gy^−1^ at a severely hypoxic condition (0.1% O_2_). The measured α values are 0.142–0.340 at an aerobic condition (21% O_2_) and have a lower value (0.036–0.080) at a severely hypoxic condition (0.1% O_2_).

## 4. Discussion

This study calculates the DNA damage induced for cells irradiated by ultrasoft X-rays and 10−15 keV X-rays. RBE values are also calculated for various oxygen concentrations, using ^60^Co γ-rays as the reference radiation type. The following sections compare the results from this study with the results obtained by experiments, track structure simulations and discuss the effect of oxygen.

### 4.1. DNA Damage Profile

The MCDS-derived RBE values for DSB induction are reported in several studies [30,78,79], including a comparison with experimental data for soft X-rays and protons [30,80,81,82,83]. The results for track structure simulations for cells irradiated with lower energy photon beams, such as 4.55 kV X-rays (*LET* = 6.57 keV/μm), show that the DNA damage profiles obtained by MCDS are up to 7% different to those derived by these track structure simulations (Table 2). All tables except Table 3 list the yield for all types of DNA damage and show that BD accounts for the greatest portion of total damage. This value decreases 3% as photon energy decreases, but other types of DNA damage increase within 2%, although to different levels (Table 1, Table 2, Table 4 and Table 5). Simple DNA damage, i.e., BD and SSBs, decreases, respectively, from 68.4% to 63.5% and 28.8% to 21.9% as photon energy decreases and complex damage, such as DSBs, increases from 1.1% to 2.0% under aerobic conditions (Table 2). This is partly attributed to the fact that a photoelectron is accompanied by correlated low-energy Auger electrons and affects the probability of forming clustered damage [84] for X-ray energies of more than ~500 eV. For lower-energy photons, the percentage of DNA damage induced by lower-energy secondary electrons increases to 30% [66]. For example, the *LET* of 100 keV electrons is low (~0.6 keV/μm) and has a long linear path (~cm) with sparse ionizations [58]. The *LET* of 5–20 keV electrons is 3.5–1.5 keV/μm. The *LET* of low-energy electrons rises dramatically from 3.5 to 12 keV/μm as energy decreases from 5 to 1 keV, with dense and clustered ionizations within a few nanometers [58]. Because GT is low-energy X-rays and produces low-energy secondary electrons, it is inferred that GT would induce more complex damage such as DSB.

In contrast to low-*LET* radiations, the portion of complex DSB for high-*LET* radiations increases to 11.9% for 2 MeV helium particles [65] (*LET* = 162.5 keV/μm [85]), so it is very difficult to repair and the cell survival rate decreases [30]. The damage is complex in the absence of oxygen. The percentage of non-DSB clustered damage composed of at least six lesions is about 9% for cells irradiated by 3.31 MeV (*LET* = 120 keV/μm) and remains 9% for an oxygen concentration of 2%. The percentage of damage composed by at least three lesions for cells irradiated by ^60^Co γ-rays decreases from 6% to 5% [86]. The RBE for cell survival under moderate hypoxia (2% O_2_) increases to a higher value than the RBE under aerobic conditions for the same LET.

### 4.2. OER and Parameters α

In Figure 2A, the OER value for DSB induction using MCDS is 2.3–2.1 as *LET* = 1~6.57 keV/μm. The other simulated OER value is ~3.0 as *LET* increases to 13 keV/μm [69].For very low-*LET* radiations, the OER values for ^60^Co γ-rays and 200 kVp X-rays, as measured using static field or pulsed field gel electrophoresis (PFGE), varies from 2.8–3.5 [17,38,87,88]. The OER values measured using the γ-H2AX foci method are almost constant at 3.0 as *LET* = 3–13 keV/μm but decrease to 2.25 at *LET* = 30 keV/μm [70]. The values measured using PFGE are 1.9–1.8 as *LET* = 6.7–20.4 keV/μm [17]. The OER value for DSB induction decreases 7% as *LET* increases from 0.24 keV/μm to 6.57 keV/μm, but the value also depends on cell type and experimental assay.

Figure 2B shows that the OER value for cell survival is 2.8–2.1, and the value decreases as *LET* increases. For a low dose, Equation (7) can be simplified to S=exp(−αD), then the OER for cell survival can be quantified as the ratio of parameter α (aerobic) to parameter α (hypoxic), i.e., $OER_{survival}=\alpha_{aerobic}/\alpha_{hypoxic}=\vartheta_{aerobic}/\theta_{hypoxic}*OER_{DSB}$. This result suggests a decreasing value of OER for DSB induction because the measured OER value for cell survival (Figure 2B) is decreasing as *LET* increases. The ratio of $\vartheta_{aerobic}/\theta_{hypoxic}$ is supposed to be larger than one. Because θ is defined as the fraction of DSBs that undergo lethal first order misrepair and damage fixation, then the presence of oxygen should increase the fraction of lethal damage due to the oxygen fixation.

Figure 3B shows that the parameter α for ultrasoft X-rays is a function of LET. The RMF model shows that the parameter *α* is linearly proportional to DSB induction when the second term can be ignored for low-*LET* radiations (see Equation (2)) and f_R_ ~1, and in that case, i.e., α=θΣ [81]. From Table 1 and a previous study [89], the DSB induction Σ is almost linearly proportional to *LET* for low-*LET* radiations at an aerobic condition (21% O_2_). Consequently, the parameter α is linearly proportional to LET. The trend of the measured parameter *α* increases as *LET* increases and can be expressed as $\alpha =\alpha_{R}+\lambda *LET$ where $\alpha_{R}$ is the *α* value for reference radiation (*LET* ~0.24 keV/μm [75]). If the parameter β (Equation (3)) is assumed to be constant with *LET* [90], i.e., $\beta \approx \beta_{R}$ (β value for reference radiation), then by using Equations (6) and (7), the RBE for GT can be expressed as [14]:

(13) $$RBE=\frac{\left(\sqrt{\alpha_{R}^{2}+4\beta_{R}D\left(\alpha_{R}+\lambda *LET+\beta_{R}D\right)}-\alpha_{R}\right)}{2\beta_{R}D}$$

where *D* is the dose of GT. In our study, the λ parameter is 0.0466 and 0.0402 μm keV^−1^ for an aerobic condition and a hypoxic condition, respectively, as shown in Figure 3B.

### 4.3. Comparison of RBE

Relative to ^60^Co γ-rays, the experimentally measured RBE values for DSB induction in cells irradiated by titanium K-shell, aluminum K-shell, copper L-shell and carbon K-shell X-rays increased from 1.4 to 2.7 as the photon energy decreased from 4.5 keV to 280 eV [17]. Other studies show that the RBE of MCDS-derived DSB yield is comparable (within 7.1%) to the experimentally measured yield of ultrasoft X-rays [34,41]. The MCDS-derived DSB yield (21.6 per Gy per Gbp) for cells irradiated with 280 eV photons in the study by Streitmatter et al. (2017) [34] gives an RBE of 2.7 relative to ^60^Co γ-rays (8.6 per Gy per Gbp). This is the same as the value that was experimentally obtained by de Lara et al. (2001) [17]. This study demonstrates that the RBE for the DSB induction of cells irradiated by 4.55 kV photons agrees well with the measured value (~1.3), as shown in Table 3.

As shown in Figure 3A, the RBE increases as the oxygen concentration decreases. That could be due to the decrease portion of indirect actions. That is, DSB induction under hypoxic conditions is a result of direct actions, as opposed to indirect actions involving O_2_. ^60^Co γ-rays are low-*LET* radiations as compared with GT and have a larger portion of DSB induction attributed to indirect actions. Therefore, RBE increases as the oxygen concentration decreases due to a larger reduction in the DSB yields induced by ^60^Co γ-rays [80]. The ratio of $\alpha /\alpha_{R}$ has been shown to be proportional to RBE [30]; however, in Figure 3B, the ratio of $\alpha /\alpha_{R}$ at 21% O_2_ is larger than the one at 0.1% O_2_ for the same *LET* value, which is opposite to the result of RBE. The experimental values of parameter α at 21% O_2_ are lower than the RMF-model-predicted values, suggesting that the RMF-model-predicted α values at 21% O_2_ may be overestimated.

A variation of RBE values is associated with the electron spectrum, which is affected by the target material and filtration. A previous study by the authors also shows that the RBE value for the DSB induction for cells irradiated by 29 kVp X-rays (molybdenum (Mo) filter) is 1.19, which is about 3% higher than the RBE value for 29 kVp X-rays (rhodium (Rh) filter), 1.16 [41]. For different biological endpoints, the RBE value for cell survival is 4.7 for cells irradiated with 29 kVp X-rays that are produced using a tungsten target and a 50 µm Rh filter. The RBE value slightly reduces to 4.4 for 29 kVp X-rays that are produced using a Mo target and a 30 µm Mo filter [19]. These results show that the variation in RBE for DSB induction is similar to that for RBE for cell survival and can be used to determine the clinical use for treatment planning. This algorithm used in this study can be used to estimate RBE values for DSB induction and cell survival for different setups, such as a different material for the target and filter.

GT is an alternative option for treating superficial tumors, such as LM and LMM in elderly patients [1,4], and is a preferred non-surgical treatment option for elderly people [5]. The energy range for GT for LM and LMM is around 9–15 kV, and the total dose prescribed is 42–160 Gy [5]. Treatment with single-fraction doses of 2 Gy and a total dose of 54–60 Gy is recommended as the standard protocol. Alternatively, the hypofractionated schedules (with a single-fraction dose of 2.5–4 Gy) are generally favored for elderly patients [5], and a dose up to 7 Gy has been reported [91]. According to Equation (13), the larger fraction dose would reduce the RBE value. For example, if the fraction dose increases from 2 Gy to 4.5 Gy, the RBE value decreases from 3% to 10% as *LET* increases from 1 keV/μm to 6.6 keV/μm.

Moreover, the higher RBE values for GT allow superficial tumors to be killed more efficiently; in other words, less dosage is required to achieve local control for a given type of tumor, especially for hypoxic tumors. If the total dose prescribed for XT is 100 Gy for aerobic cells, then it may require a total dose up to 300 Gy for hypoxic tumors (OER ~3 [72]) because the oxygen concentration in the tumor cells is significantly lower [92]. The RBE value for GT is 1.2–2.4 for severely hypoxic tumors, and it suggests the dose for GT can be as low as half to the total dose prescribed for XT and can reduce the complications. Further studies could use a combination of nanoparticles to increase the dose [93] and the synergistic effects could further improve the outcomes of clinical treatment.

## 5. Conclusions

The MCDS-derived RBE values for Grenz rays for DSB induction are 1.1–1.3, and the RBE increases as the photon energy decreases (and *LET* increases). The change in the OER for DSB induction is insignificant as *LET* increases; however, the OER for cell survival increases as *LET* increases. This study thus shows that GT results in more DNA damage induction and allows more effective tumor control for superficial tumors, as indicated by the OER value and the RBE for DSB induction and cell survival.

## Figures and Tables

**Table 1:** Comparison MCDS results (mean ± standard error) for monoenergetic electrons and those obtained by track structure simulations.

MCDS	Track Structure ^b^	*LET*^e^ (keV/μm)	BD ^e^ (%)	SSB ^e^ (%)	SSB^+^^e^ (%)	2SSB ^e^ (%)	DSB ^e^ (%)	DSB^+^^e^ (%)	DSB^++^^e^ (%)	Total SSB ^e^ (Gy^−1^ Gbp^−1^)	Total DSB ^e^ (Gy^−1^ Gbp^−1^)
100 eV ^d^		21.47	44.1	32.9	8.3	3.77	4.72	3.19	3.02	102.4 ± 0.1	24.9 ± 0.1
	100 eV	21.47	73.9	22.4	1.9	0.09	1.39	0.27	0.02	162.5	9.8
300 eV ^d^		16.95	55.2	33.0	4.9	1.52	3.33	1.42	0.65	139.5 ± 0.1	19.1 ± 0.1
	300 eV ^a^	16.95	66.4	26.6	3.3	0.43	2.38	0.85	0.09	162.5	15.0
500 eV ^d^		14.12	59.5	32.1	3.6	0.92	2.67	0.88	0.29	154.2 ± 0.1	16.1 ± 0.1
	500 eV ^b^	14.12	68.7	24.4	2.8	0.47	1.86	0.79	0.07	162.5	13.0
1000 eV ^d^		10.28	63.5	30.9	2.5	0.50	2.00	0.48	0.11	168.6 ± 0.1	12.9 ± 0.1
	1000 eV ^b^	10.28	68.9	25.2	2.8	0.50	1.81	0.71	0.08	156.0	13.0
1500 eV ^d^		8.25	65.1	30.3	2.1	0.37	1.74	0.36	0.07	174.3 ± 0.1	11.5 ± 0.1
	1500 eV ^a^	8.25	70.5	24.3	2.4	0.40	1.69	0.63	0.07	156.0	11.7
4500 eV ^c^		4.08	66.2	29.9	1.8	0.20	1.60	0.30	0.05	123.5	7.7
	4500 eV ^a^	4.08	71.4	24.1	2.1	0.29	1.47	0.55	0.04	123.5	7.8

^a^ The data are from the published data of Nikjoo et al. [64,65]. SSB and DSB yields are converted into the units of Gy^−1^ cell^−1^ using a factor 3.9 × 10^12^ Da cell^−1^. ^b^ The data are from the published data of Nikjoo et al. (1997) [66]. ^c^ The data are from the published data of Semenenko and Stewart (2004) [50]. ^d^ All values for standard errors of the percentages of BD, SSB, SSB^+^, 2SSB, DSB, DSB^+^ and DSB^++^ obtained by this work are less than 0.03%. The *p* values of the comparisons in SSB and DSB yields between any two groups are less than 0.01. ^e^ LET: linear energy transfer; MCDS: Monte Carlo damage simulation; BD: base damage; SSB: single-strand break; DSB: double-strand break.

**Table 2:** Relative yield of DNA damage and absolute yields and the values for standard error of DSB and SSB (per Gy per Gbp) induced by X-rays and ^60^Co γ-rays at a normal oxygen concentration (21%).

Photon Energy	BD ^d^ (%)	SSB ^d^ (%)	SSB^+^^d^ (%)	2SSB ^d^ (%)	DSB ^d^ (%)	DSB^+^^d^ (%)	DSB^++^^d^ (%)	Total SSB ^d^ (Gy^−1^ Gbp^−1^)	Total DSB ^d^ (Gy^−1^ Gbp^−1^)
Monoenergetic 15 kV (15 kV ^a,c^)	66.9	29.4	1.6	0.3	1.4	0.3	0.1	184.5 ± 0.1	8.9 ± 0.1
Monoenergetic 10 kV (10 kV ^a,c^)	66.0	29.7	1.9	0.4	1.6	0.3	0.1	182.7 ± 0.1	9.3 ± 0.1
10 kVp (5.7 kV ^a,c^)	64.4	30.3	2.3	0.5	1.8	0.5	0.2	179.1 ± 0.1	10.2 ± 0.1
4.55 kV (4.55 kV ^a,c^)	63.5	30.5	2.6	0.6	2.0	0.6	0.2	176.9 ± 0.1	10.8 ± 0.1
4.55 kV ^b^	75.2	21.9	1.3	0.4	0.9	0.2	0.0	-	-
^60^Co (1250 kV ^a,c^)	68.4	28.8	1.3	0.2	1.1	0.2	0.0	187.4 ± 0.1	8.1 ± 0.1

^a^ Averaged photon energy. ^b^ The DNA profile was derived from the previous study [68]. ^c^ All values for standard errors of the percentages of BD, SSB, SSB^+^, 2SSB, DSB, DSB^+^ and DSB^++^ obtained by this work are less than 0.1%. The *p* values of the comparisons in SSB and DSB yields between any two groups are less than 0.01. ^d^ BD: base damage; SSB: single-strand break; DSB: double-strand break.

**Table 3:** Absolute yield of DSBs (per Gy per Gbp) (mean ± standard error) induced by soft X-rays and ^60^Co γ-rays at an oxygen concentration of 21%.

Photon Energy	*LET*^c^ (keV/μm)	Measured DSBs ^c^ (per Gbp per Gy)^b^	RBE ^c^	MCDS ^c^ DSB ^c^ s (per Gbp per Gy)	RBE ^c^
Monoenergetic 15 kV (15 kV ^a^)	2.7	-	-	8.9 ± 0.1	1.1 ± 0.0
Monoenergetic 10 kV (10 kV ^a^)	3.7	-	-	9.3 ± 0.1	1.2 ± 0.0
10 kVp (5.7 kV ^a^)	5.6	-	-	10.2 ± 0.1	1.3 ± 0.0
4.55 kV (4.55 kV ^a^)	6.6	10.4 ^a^	1.4	10.8 ± 0.1	1.3 ± 0.0
^60^Co (1250 kV ^a^)	0.24	7.6 ^a^	1.0	8.1 ± 0.1	-

^a^ Averaged photon energy. ^b^ The DSB yield is derived from a previous study [17]. ^c^ LET: linear energy transfer; MCDS: Monte Carlo damage simulation; DSB: double-strand break; RBE: relative biological effectiveness.

**Table 4:** Relative yield of DNA damage and absolute yield of DSB and SSB (per Gy per Gbp) (mean ± standard error) at a hypoxic oxygen concentration of 2%).

Photon Energy	BD ^c^ (%)	SSB ^c^ (%)	SSB^+^^c^ (%)	2SSB ^c^ (%)	DSB ^c^ (%)	DSB^+^^c^ (%)	DSB^++^^c^ (%)	Total SSB ^c^ (Gy^−1^ Gbp^−1^)	Total DSB ^c^ (Gy^−1^ Gbp^−1^)
Monoenergetic 15 kV (15 kV ^a,b^)	67.6	29.1	1.5	0.2	1.3	0.2	0.1	171.1 ± 0.1	7.5 ± 0.1
Monoenergetic 10 kV (10 kV ^a,b^)	66.8	29.4	1.7	0.3	1.4	0.3	0.1	169.7 ± 0.1	7.9 ± 0.1
10 kVp (5.7 kV ^a,b^)	65.2	30.0	2.1	0.4	1.7	0.4	0.2	166.8 ± 0.1	8.7 ± 0.1
4.55 kV (4.55 kV ^a,b^)	64.4	30.3	2.3	0.5	1.8	0.5	0.2	165.0 ± 0.1	9.2 ± 0.1
^60^Co (1250 kV ^a,b^)	69.0	28.5	1.2	0.2	1.0	0.1	0.0	173.5 ± 0.1	6.9 ± 0.1

^a^ Averaged photon energy. ^b^ All values for standard errors of the percentages of BD, SSB, SSB^+^, 2SSB, DSB, DSB^+^ and DSB^++^ obtained by this work are less than 0.1%.The *p* values of the comparisons in SSB and DSB yields between any two groups are less than 0.01. ^c^ BD: base damage; SSB: single-strand break; DSB: double-strand break.

**Table 5:** Relative yield of DNA damage and absolute yield of DSB and SSB (per Gy per Gbp) (mean ± standard error) at a hypoxic oxygen concentration of 0.1%).

Photon Energy	BD ^c^ (%)	SSB ^c^ (%)	SSB^+^^c^ (%)	2SSB ^c^ (%)	DSB ^c^ (%)	DSB^+^^c^ (%)	DSB^++^^c^ (%)	Total SSB ^c^ (Gy^−1^ Gbp^−1^)	Total DSB ^c^ (Gy^−1^ Gbp^−1^)
Monoenergetic 15 kV (15 kV ^a,b^)	69.8	28.1	1.0	0.1	0.9	0.1	0.0	126.0 ± 0.1	3.8 ± 0.1
Monoenergetic 10 kV (10 kV ^a,b^)	69.1	28.3	1.2	0.2	1.0	0.2	0.0	125.4 ± 0.1	4.1 ± 0.1
10 kVp (5.7 kV ^a,b^)	67.8	28.8	1.5	0.3	1.2	0.3	0.1	124.3 ± 0.1	4.6 ± 0.1
4.55 kV (4.55 kV ^a,b^)	67.1	29.2	1.7	0.3	1.4	0.3	0.1	123.8 ± 0.1	4.9 ± 0.1
^60^Co (1250 kV ^a,b^)	70.9	27.5	0.8	0.1	0.7	0.1	0.0	127.0 ± 0.1	3.5 ± 0.1

^a^ Averaged photon energy. ^b^ All values for standard error of the percentages of BD, SSB, SSB^+^, 2SSB, DSB, DSB^+^ and DSB^++^ obtained by this work are less than 0.5%. The *p* values of the comparisons in SSB and DSB yields between any two groups are less than 0.05. ^c^ BD: base damage; SSB: single-strand break; DSB: double-strand break.

## Data Availability

The data presented in this study are available in this article.
